# Interstitial Keratuis

**Published:** 1892-07-16

**Authors:** 


### OPHTHALMOLOGY.

### INTERSTITIAL KERATITIS.

Interstitial Keratitis is a chronic inflammation of the cornea, consisting of changes in the corneal tissue with the formation of blood vessels in its deeper layers, the cornea becomes hazy and cloudy, and, eventually, a large amount of opacity may be the result, so that the deeper structures of the eye may be obscured from sight, and great interference with vision takes place. It generally occurs in those who are the subject of hereditary syphilis or of struma.

It may be so slight that the unaided sight fails to detect it, but even then there will be considerable interference with correct vision, which can be ascertained by testing for near and far sight with the test types, when it will be found there is a considerable departure from the normal acuity. If the eye be then examined by a bright light by aid of the method of oblique illumination a cloudiness of the cornea will be readily demonstrated, and it will be found that the fundus is partially obscured by both the direct and indirect methods of ophthalmoscopy. There may, however, be considerable local congestion of the vessels around the cornea, and vessels may be seen ramifying in its structure, while it may exhibit a "ground glass" appearance, believed by some to be pathognomonic of hereditary syphilis, and in these cases the diagnosis may be generally confirmed by the appearance of the teeth, the crescentically notched upper incisors, with malformation of the other incisors, so that they appear peg-shaped and separated by intervals from one another instead of lying in close apposition, as pointed out by Hutchinson. Other symptoms of the syphilitic taint may also be present, as the puckered lines around the mouth, and the family history of repeated abortion on the part of the mother, and ill-health of the surviving children.

There may be no such appearances, but the patients exhibit the characteristic appearance of the strumous diathesis, while in some neither taint is manifested. The course of the complaint is generally chronic, extending over many months, and while considerable improvement may be expected in time from appropriate treatment, in a large number of cases opacities of the cornea remain, rendering the eye practically useless and much disfigured. It is therefore necessary to give a very guarded prognosis. It generally occurs before puberty, but sometimes comes on in adult life. In some cases it takes the form of a diffuse keratitis, while in others a portion of the margin is chiefly affected, having a dull reddish appearance, known as the "salmon patch," from the formation of blood vessels. The treatment consists in that of the constitutional affection. In syphilis, by the inunction of mercury, this may be carried out in young children by rubbing mercurial ointment, a piece about the size of a pea, into the axillæ at bedtime, or by wrapping a flannel bandage smeared with the ointment round the abdomen at night, the gums being carefully watched so that salivation be not produced, or grey powder may be administered by the mouth in small doses at frequent intervals, half a grain every fourth hour. No stimulating application must be applied to the eye, but weak atropine drops may be instilled to allay irritation when it is present, and a shade worn to diminish the intensity of the photophobia, which, however, is not always present. In those who have previously had a course of mercury the iodide of potassium may be administered with advantage. In the later stages the opacities may be lessened by gentle frictions with the yellow oxide of mercury to the cornea.

In the strumous cases tonics, especially iron, quinine, and iodine, and cod-liver oil should be given; change to sea air is very beneficial, while generous diet of wholesome, nourishing food must not be forgotten, this disease often occurring in those who are underfed and in the poorer walks of life.

